# Low skeletal muscle mass predicts frailty in elderly head and neck cancer patients

**DOI:** 10.1007/s00405-021-06835-0

**Published:** 2021-05-06

**Authors:** C. D. A. Meerkerk, N. Chargi, P. A. de Jong, F. van den Bos, R. de Bree

**Affiliations:** 1 Department of Head and Neck Surgical Oncology, Division of Imaging and Oncology Center, University Medical Center Utrecht, Heidelberglaan 100, 3584 CX Utrecht, The Netherlands; 2 Department of Radiology. Division of Imaging and Oncology, University Medical Center Utrecht and Utrecht University, Heidelberglaan 100, 3584 CX Utrecht, The Netherlands; 3 Department of Geriatric Medicine, University Medical Center Utrecht, Heidelberglaan 100, 3584 CX Utrecht, The Netherlands; 4 Department of Head and Neck Surgical Oncology, University Medical Center Utrecht, House Postal Number Q.05.4.300, PO BOX 85500, 3508 GA Utrecht, The Netherlands

**Keywords:** Sarcopenia, Comprehensive Geriatric Assessment, Muscle function, Handgrip strength, Skeletal muscle index

## Abstract

**Purpose:**

Treatment of head and neck cancer (HNC) carries a high risk of adverse outcomes in patients, especially in frail elderly. Therefore, it is important to identify patients in which treatment benefits outweigh the risk of any adverse outcome. Although the comprehensive geriatric assessment (CGA) identifies frailty, it is a time-consuming tool. Instead, measurement of skeletal muscle mass and strength (sarcopenia) may be a promising and time-efficient biomarker for frailty. The aim of this study was to examine the association between sarcopenia and frailty assessment tools, such as the CGA, Fried criteria and the Groningen Frailty Indicator (GFI).

**Methods:**

A retrospective study was performed in elderly patients (≥ 70-years) with HNC. Sarcopenia was defined as the combination of reduced handgrip strength (HGS) and low skeletal muscle mass (SMM), according to the EWGSOP-2 criteria. SMM was measured on routinely available diagnostic imaging and corrected height: skeletal muscle index (SMI). A CGA was performed by a geriatrician. Frailty screening was performed using the GFI and the Fried criteria.

**Results:**

In total, 73 patients were included of which 33 were men (45.2%) and 40 women (54.8%). Frail patients diagnosed by CGA were more likely to have low SMI, sarcopenia, more comorbidities and were at high risk for malnutrition (all *p* < 0.05). In multivariate regression analysis, the only significant predictor for frailty diagnosed by CGA was SMI (OR 0.9, *p* < 0.01) independent of comorbidity and muscle strength.

**Conclusion:**

Low SMI and sarcopenia are associated with frailty in elderly HNC patients. Low SMI predicts frailty and is a promising time-efficient and routinely available tool for clinical practice.

## Introduction

Head and neck cancer (HNC) is among the most frequent malignant tumors in the world with an annual incidence of more than 650,000 cases and 330,000 deaths [1]. Of these patients, more than 60% have an age at diagnosis of 60 years or more [2]. With the global aging of the worldwide population, it is to be expected that the incidence of HNCs will increase. Besides advanced age, a significant amount of pre-existent comorbidities in HNCs patients is an additional negative prognostic factor that reduces overall survival [3].

Treatment of HNCs is often complex and requires, based on tumor-specific and patient-specific characteristics, surgery with or without adjuvant (chemo)radiotherapy or radiotherapy with or without chemotherapy with salvage surgery in reserve for residual or recurrent loco regional disease [4]. These treatments are effective, but have significant risk of toxicities, complications, and even mortality [5]. Treatment could also decrease quality of life, for instance speech problems, fatigue or trouble with social eating caused by dry mouth, and swallowing problems [6, 7].

Due to the growing incidence of both HNCs worldwide and the global aging of the population, it is of great importance to identify key predictive and prognostic factors for treatment outcomes in older patients with HNC. This knowledge can be useful for clinicians and patients in (shared) decision-making weighing suitability of treatment, prognosis, and expected quality of life. Although this knowledge is also important in younger HNC patients, it is even more warranted in older HNC patients due to their vulnerability, decreased physical and mental compensation mechanisms compared to younger patients. This vulnerability is also being referred to as frailty.

A comprehensive geriatric assessment (CGA) is the most appropriate way to detect frailty [8]. A CGA is a multidisciplinary, multidimensional, and systematic assessment, and consists of validated scales to identify impairments in the four geriatric domains: somatic, functional, nutritional, and psychosocial [8]. Frailty is associated with poor treatment outcomes and health‐related quality of life [7]. Because performing CGA is time-consuming and not all patients will benefit from a CGA, screening methods have been developed to identify those at risk for adverse health outcomes and who may benefit from a CGA. However, the available frailty screening methods may have insufficient discriminative power to select patients for further assessment [9].

Sarcopenia also frequently observed in older patients is suggested as a more reliable, inexpensive and easy alternative for frailty screening questionnaires in HNC patients [10]. However, there is much discussion on different definitions of frailty and sarcopenia [11]. By the European Working Group on Sarcopenia in Older People (EWGSOP-2), sarcopenia is described as a generalized and progressive loss of muscle function (MF) and skeletal muscle mass (SMM), caused by adverse muscle changes that accrue across a lifetime [12]. Sarcopenia itself is also related with adverse health outcome, such as chemotherapy dose-limiting toxicity [13], increased incidence of postoperative complications, and decreased survival [14, 15].

The relation between low skeletal muscle mass, measured using CT of the head and neck, and frailty screening methods was recently reported by Zwart et al. [10]. However, the direct relation of sarcopenia and CGA, as gold standard for frailty, has yet to be determined.

Therefore, the aim of this study was to examine the association between sarcopenia, defined as the combination of low muscle strength and low muscle mass, and frailty, diagnosed by CGA. Our secondary aim was to examine the association between sarcopenia and the frailty Fried criteria and the Groningen Frailty Indicator (GFI) frailty screening test.

## Materials and methods

### Ethical approval

The design of this study was approved by the Medical Ethical Research Committee of the University Medical Center Utrecht (approval ID 17–365/C). All procedures in this study were in accordance with the ethical standards of the institutional and/or national research committee and with the 1964 Helsinki declaration (Version 2008) and its later amendments or comparable ethical standards. All data were handled according to general data protection regulation (GDPR).

### Patients and study design

This study was designed as a single-center retrospective study. Older patients (≥ 70 years old) with pathologically proven head and neck squamous cell carcinoma (HNSCC) treated between April 2015 and February 2018 of whom results of a CGA, Fried Frailty criteria, GFI screening questionnaire, and pre-treatment CT or MRI during their diagnostic work-up were available, were included in this study. In this period, at our center, elderly HNSCC patients were offered geriatric assessment, but patients could refuse. As a consequence, not all older patients underwent frailty assessment at that time. Histologic tumor types other than squamous cell carcinoma were excluded.

Relevant demographic and clinical variables were collected from patient’s medical record: age at diagnosis, sex, body mass index (BMI), percentage of weight loss in 6 months prior to diagnosis, smoking status, alcohol use, nutritional status at diagnosis as evaluated by the Malnutrition Universal Screening Tool (MUST), comorbidities as evaluated by the Charlson comorbidity index (CCI), localization of the tumor, tumor type (primary, second primary or recurrence), and the TNM stage according the 8th edition of the UICC tumor classification of malignant tumors.

### Sarcopenia

#### Definition of sarcopenia

Sarcopenia was defined as the combination of low MF, as determined by muscle strength, and low muscle quantity, as determined by SMM, according to the recommendation by the EWGSOP-2 and further explained below [12].

#### Muscle function: muscle strength

Overall muscle strength is strongly related with isometric handgrip strength (HGS) [16]. HGS was measured using a Jamar hydraulic handheld dynamometer according to the recommendations of the American society of hand therapist’s (ASHT) and expressed in kilograms (kg). Patients were asked to squeeze maximally with each hand. The average score of the left and right hands was used for analysis. Patients had low HGS if the mean HGS was below 27 kg (men) or below 16 kg (women) [12].

#### Skeletal muscle mass

**Fig. 1:**
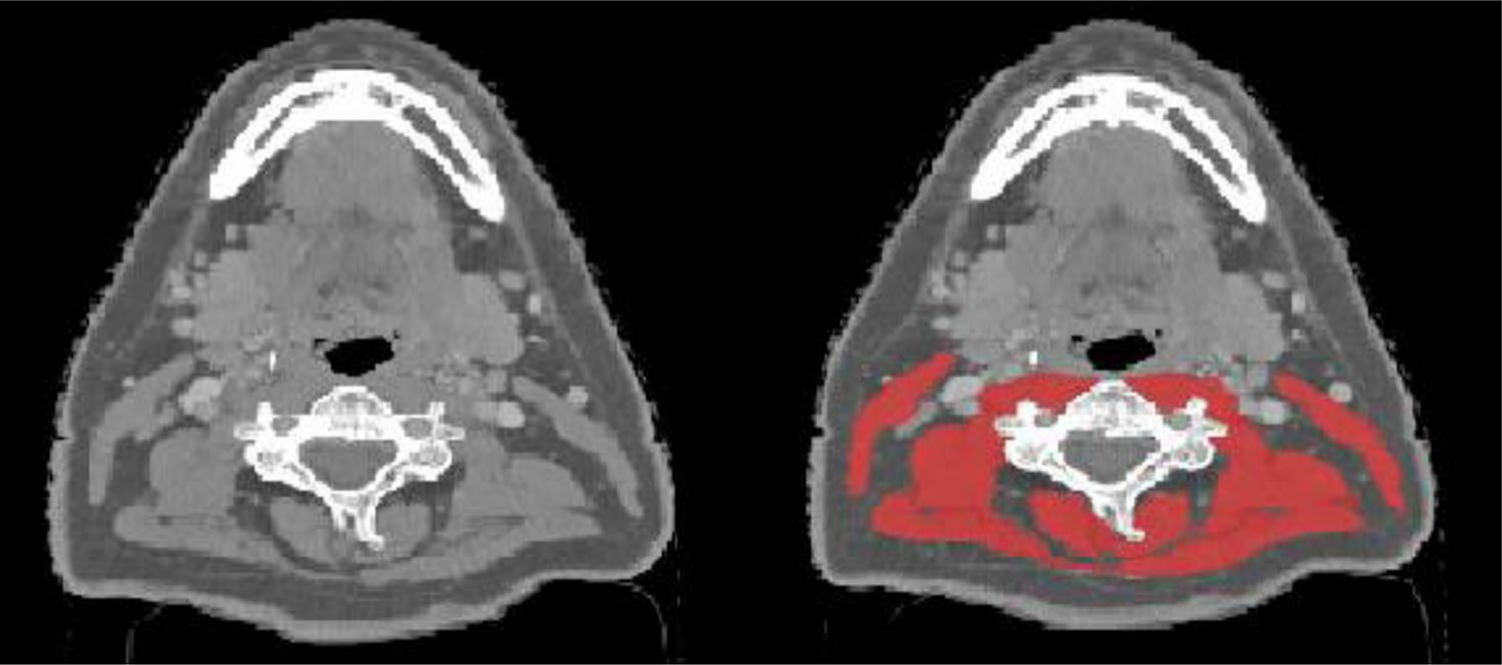
Example of segmentation of skeletal muscle tissue at the level of the third cervical vertebra (C3). Two identical axial contrast enhanced computed tomography (CT) slides at the level of C3; left shows the muscle tissue unsegmented, right shows both sternocleidomastoid and paravertebral muscles segmented in red

SMM was measured as cross-sectional muscle area (CSMA) on pretreatment CT or MRI imaging of the head-and-neck area at the level of the third cervical vertebrae (C3). The axial slide of the imaging which showed both transverse processes and the entire vertebral arc was selected for the segmentation of muscle tissue. For CT imaging, muscle area was defined as the pixel area between the radio density range of − 29 and + 150 Hounsfield units (HU), which is specific for muscle tissue [17]. For MRI, muscle area was manually segmented, and fatty tissue was manually excluded (Fig. 1).

Segmentation of muscle tissue was manually performed using the commercially available software package SliceOmatic (version 5.0, Tomovision, Canada) by a single researcher (C.M.) who was blinded for outcome regarding frailty and sarcopenia. Cross-sectional muscle area at the level of C3 was converted to CSMA at the level of L3 using a previously published formula [18]. The lumbar skeletal muscle index (SMI) was calculated by correcting SMM at the level of L3 for height. Patients had a low SMI if this value was below 43.2 cm^2^/m^2^; this cut-off value was established in a separate cohort of patients with head-and-neck cancer [13].

### Comprehensive geriatric assessment (CGA)

The CGA conducted in this study consists of four domains; the somatic, psychological, functional and social domains and was performed by a geriatrician. Specific, validated tools per geriatric domain were used. For the somatic domain, the Charlson Comorbidity Index (CCI)[19], the Malnutrition Universal Screening Tool (MUST) [20], and polypharmacy are used. The psychological domain was examined by the Mini Mental State Examination (MMSE) [21] for cognitive function and Geriatric Depression Scale (GDS) [22] for depression. For the functional domain, activities of daily living (ADL) were examined with The Katz Activities of Daily Living (KATZ-6) [23] and KATZ-9 was used for scoring Instrumental ADL [24]. Social status was determent on questions about current living situation, social activities, presence of informal care system/social support. Each instrument was defined as abnormal according to validated cut-off scores. The cut-off scores are listed in Table 1. Overall, a patient was considered frail if the CGA had an abnormal outcome on at least two of the instruments used.

**Table 1:** Overview of the selected screening instruments for CGA

Geriatric domain		Measure	Score range or (cut-off)
Somatic	Comorbidity	*CCI*	0–31
	Nutrition	*MUST*	0–3 (≥ 2)
	Medication	*-*	Ordinal (> 4)
Psychological	Cognition	*MMSE*	0–30 (≤ 24)
	Depression	*GDS-2 or*	0–2 (≥ 1)
		*GDS 15*	0–15 (≥ 6)
Functional	Function	*ADL KATZ*	0–6, (≥ 1)
		*IADL KATZ*	0–9, (≥ 1)
Social		Living situation, social activities and informal care system	0–3 (≥ 2)

*ADL* activities of daily living, *IADL* instrumental activities of daily living, *MMSE* mini-mental state exam, *GDS* geriatric depression scale, *MUST* malnutrition universal screening tool, *CCI* charlson comorbidity index

### Fried frailty criteria

The Fried Frailty criterion is an operational definition of physical frailty based on the presence of three or more of the following five criteria: unintentional weight loss, exhau stion, low physical activity level, slow gait speed, and low handgrip strength [25]. In older patients with cancer, the sensitivity and specificity of the Fried frailty criteria for predicting frailty, based on CGA, are amongst 25–37% and 86–96%, respectively [9]. The Fried frailty criteria are known to be useful in predicting complications, length of hospital stay and other adverse health outcome in patients with HNC [26].

### Groningen frailty indicator

GFI is a 15-item frailty screening tool to evaluate frailty status in geriatrics through loss of function and resources in physical, social, and psychological domains. Patients were categorized as non-frail (GFI < 4) and frail (GFI ≥ 4) [27]. In older patients with cancer the sensitivity and specificity of the GFI for predicting frailty, based on CGA, are amongst 39–62% and 69–87%, respectively [9]. The GFI is also useful in predicting postoperative complications, however, this questionnaire is not specially designed for oncological patients [26].

### Statistical analysis

Data analyses were performed using IBM SPSS statistics 25. First, the patient cohort was described regarding the baseline. Continuous data are represented as mean ± standard deviation (SD). Categorical data are represented as a number and percentage of total.

MF was presented dichotomously as low MF and normal MF based on previously published gender specific cut-offs for HGS. The SMM, was presented dichotomously as low SMI and normal SMI based on previously published specific cut-offs for SMI. Sarcopenia was presented dichotomously as sarcopenic (only if patients had a low HGS and low SMI) and non-sarcopenic (all other patients).

Frailty was presented dichotomously as frail and non-frail based on abovementioned and previously published cut-offs for frailty based on the CGA, Fried criteria or GFI. Independent sample t tests or Chi-square statistics were used for analyzing differences between the frequencies of each categorical variable with the presence or absence of sarcopenia and presence or absence of frailty.

Univariate logistic regression analyses were performed, with sarcopenia or frailty as dependent variable and the baseline variables as independent variables. Variables were selected based on clinical relevance by exploring literature. Variables that were statistically significant (α < 0.05) in the univariate regression were included in the multivariate logistic regression. In this way, odds ratios (ORs) and 95% CIs were provided.

## Results

In total, 85 patients were referred to the geriatrician in the inclusion period. Because 12 patients did not undergo full CGA or Fried Frailty criteria or GFI screening, questionnaires were not complete, finally 73 patients were included. The mean age was 81.73 (6.24 SD). The majority of the patients was female (55%). The mean BMI was 26.80 (5.70 SD) and most of the patients did not report loss of weight 6 months prior to diagnosis (63%). The majority of the patients used alcohol (56%) and were former smokers (55%). Most patients had a high CCI comorbidity score of > 6 (63%). According to the TNM classification, most patients had stage IV disease (44%).

Of the included 73 patients, 33 (45%) patients had low muscle strength, 58 (79%) had low SMI. A total of 24 (33%) patients were defined as sarcopenic. Based on the CGA, 39 (54%) patients were defined as frail. Based on the frailty Fried criteria, 21 (29%) patients were defined as frail, as the GFI defined 38 (52%) patients frail. An overview of the characteristics of patients is listed in Table 2**.**

**Table 2:** Characteristics of patients with and without sarcopenia

	Total		Sarcopenic		Non sarcopenic		*χ*^2^	*p *value
	*N* = 73		*N* = 24		*N* = 49			
Age (years) (M, SD)	81.73	6.24(SD)	83.7	5.73(SD)	80.24	6.21(SD)	NA	0.025
Sex (*n*, %)								
Male	33	45	10	42	23	47	0.181	0.671
Female	40	55	14	58	26	53		
Weight loss 6 months prior to diagnosis (n, %)								
Non	46	63	15	63	31	63	0.392	0.822
< 10%	20	27	6	25	14	29		
≥ 10%	7	10	3	13	4	8		
BMI (kg/m^2^)	26.80	5.70(SD)	25.12	4.99(SD)	27.92	4.49(SD)	NA	0.018
Smoker (*n*, %)								
No	25	34	11	46	14	29	2.604	0.272
Former	40	55	10	42	30	61		
Current	8	11	3	13	5	10		
MUST score (*n*, %)								
0	53	72	18	75	35	71	0.533	0.766
1	1	2	0	0	1	2		
2	19	26	6	25	13	27		
Alcohol use (*n*, %)								
No	25	34	9	38	16	33	1.340	0.720
Yes	41	56	14	58	27	55		
Former	7	10	1	4	6	12		
Charlson comorbidity index (*n*, %)								
Low ≤ 6	27	37	6	25	21	43	2.204	0.138
High > 6	46	63	18	75	28	57		
Localization (*n*, %)								
Oral cavity	46	63	14	58	32	65	12.284	0.584
Nasopharynx	2	3	1	4	1	2		
Oropharynx	2	3	0	0	2	4		
Hypopharynx	3	4	1	4	2	4		
Larynx	7	10	2	8	5	10		
Skin	8	11	3	13	5	10		
Paranasal sinuses	2	2	1	4	2	4		
Type of tumor (*n*, %)								
Primary	56	77	17	71	39	80	1.019	0.601
Recurrent	11	15	4	17	7	14		
Second primary	6	8	3	13	3	6		
TNM Stage (*n*, %)								
I	8	11	5	21	3	6	3.708	0.295
II	19	26	5	21	14	29		
III	14	19	4	17	10	20		
IV	32	44	10	42	22	45		
Low Muscle strength (*n*, %)								
No	40	55	0	0	40	82	43.340	0.000
Yes	33	45	24	100	9	18		
Low SMI (*n*, %)								
No	15	21	0	0	15	31	9.247	0.002
Yes	58	79	24	100	34	69		
Frailty Fried criteria (*n*, %)								
No	52	71	10	42	42	86	15.253	0.000
Yes	21	29	14	58	7	14		
Frailty GFI (*n*, %)								
No	35	50	9	38	26	53	1.563	0.211
Yes	38	52	15	63	23	47		
Frailty CGA (*n*, %)								
No	34	46	7	29	27	55	4.355	0.037
Yes	39	54	17	71	22	45		

*BM* body mass index, *MUST* malnutrition universal screening tool, *SMI* skeletal muscle index, *GFI* groningen frailty indicator, *CGA* comprehensive geriatric assessment

### Correlations

Table 2 shows statistically significant differences in CGA, Fried criteria, age at diagnosis, and BMI between patients with and without sarcopenia. Patients with sarcopenia were more likely to be frail according to the CGA (71% versus 45%; *p* < 0.05) and the Fried criteria (58% versus 14%; *p* < 0.00), to be older of age (mean 83.7 years versus 80.24 years; *p* < 0.05), and to have a lower BMI at diagnosis (25.12 versus 27.92, *p* < 0.05).

Table 3 shows statistically significant differences in sarcopenia, age at diagnosis, sex, low SMI, Fried criteria, and GFI between patients with and without frailty, diagnosed with CGA. Frail patients were more likely to be sarcopenic (44% versus 21%, *p* < 0.05), to be older of age (mean 83.5 years versus 79.0 years; *p* < 0.05), to be female (69% versus 38%, *p* < 0.05), to have a low SMI at diagnosis (90% versus 68%, *p* < 0.05), to be frail according to the Fried criteria (49% versus 6%; *p* < 0.00), and the GFI (77% versus 24%; *p* < 0.00).

**Table 3:** Characteristics of patients with and without frailty based on the CGA

	Total		Frail		Non frail		***χ***^**2**^	***p ***value
	*N* = 73		*N* = 39		*N* = 34			
Age (years) (M, SD)	81.73	6.24(SD)	83.49	6.47(SD)	78.96	5.03(SD)	NA	**0.002**
Sex (*n*, %)								
Male	33	45	12	31	21	62	7.045	**0.008**
Female	40	55	27	69	13	38		
Weight loss 6 months prior to diagnosis (*n*, %)								
Non	46	63	22	56	24	71	1.839	0.399
< 10%	20	27	12	31	8	24		
≥ 10%	7	10	5	13	2	6		
BMI (kg/m2)	26.80	5.70(SD)	26.98	5.66(SD)	27.02	3.69(SD)	NA	0.970
Smoker (*n*, %)								
No	25	34	15	38	10	29	3.072	0.215
Former	40	55	18	46	22	65		
Current	8	11	6	15	2	6		
MUST score (*n*, %)								
0	53	72	24	62	29	85	7.533	**0.023**
1	1	2	0	0	1	3		
2	19	26	15	38	4	12		
Alcohol use (*n*, %)								
No	25	34	15	38	10	29	1.498	0.683
Yes	41	56	21	54	20	59		
Former	7	10	3	8	4	12		
Charlson comorbidity index (*n*, %)								
Low ≤ 6	27	37	10	26	17	50	4.624	**0.032**
High > 6	46	63	29	74	17	50		
Localization (*n*, %)								
Oral cavity	46	63	27	69	19	56	17.392	0.236
Nasopharynx	2	3	1	3	1	3		
Oropharynx	2	3	1	3	1	3		
Hypopharynx	3	4	0	0	3	9		
Larynx	7	10	2	5	5	15		
Salivary glands	3	4	2	5	1	3		
Skin	8	11	5	13	3	9		
Paranasal sinuses	2	2	1	3	1	3		
Type of tumor (*n*, %)								
Primary	56	77	28	72	28	82	5.776	0.056
Recurrent	11	15	5	13	6	18		
Second primary	6	8	6	15	0	0		
TNM Stage (*n*, %)								
I	8	11	5	13	3	9	1.047	0.790
II	19	26	11	28	8	24		
III	14	19	6	15	8	24		
IV	32	44	17	44	15	44		
Low muscle strength (*n*, %)								
No	40	55	20	51	20	59	0.417	0.518
Yes	33	45	19	49	14	41		
No	15	21	4	10	11	32	5.432	**0.020**
Yes	58	79	35	90	23	68		
Frailty fried criteria (*n*, %)								
No	52	71	20	51	32	94	16.265	**0.000**
Yes	21	29	19	49	2	6		
Frailty GFI (*n*, %)								
No	35	50	9	23	26	76	20.749	**0.000**
Yes	38	52	30	77	8	24		
Sarcopenia (*n*, %)								
No	49	67	22	56	27	79	4.355	**0.037**
Yes	24	33	17	44	7	21		

*BMI* Body mass index, *MUST* Malnutrition universal screening tool, *SMI* skeletal muscle index, GFI groningen frailty indicator, *CGA* comprehensive geriatric assessment

### Univariate and multivariate logistic regressions analyses

Table 4 shows the univariate and multivariate logistic regression analyses with sarcopenia as the dependent variable. The univariate regression analysis with sarcopenia as dependent variables distinguished age at diagnosis (OR 3.39, 95% CI 1.51–9.99, *p* = 0.027), BMI (OR 0.87, 95% CI 0,78–0,98, *p* = 0.024), frailty according CGA (OR 2.98, 95% CI 1.05–8.47, *p* = 0.040), and frailty according Fried criteria (OR 1.92 95% CI 1.28–2.87, *p* = 0.002) as significant variables for predicting sarcopenia. These significant variables were subjected to two different multivariate analyses: the first with frailty CGA, and the second with frailty Fried criteria because of assumed multicollinearity. In the first multivariate analysis, only BMI (OR 0.87, 95% CI 0.77–0.98, *p* = 0.022) remained significant. In the second age at diagnosis (OR 3.59, 95% CI 1.02–12.58, *p* = 0.046), frailty Fried criteria (OR 1.89, 95% CI 1.22–2.93, *p* = 0.004), and BMI (OR 0.87, 95% CI 0.76–0.99, *p* = 0.033) remained significant.

**Table 4:** Univariate and multivariate logistic regression analysis for analyzing variables associated with sarcopenia in HNC patients

Sarcopenia	Univariate analysis			Multivariate analysis					
	OR	95% CI	*p* value	OR	95% CI	*p* value	OR	95% CI	*p* value
Age (years)									
≤ 80	Ref			Ref			Ref		
> 80	3.391	1.51–9.99	0.027**	3.196	0.98–10.34	0.052	3.587	1.02–12.58	0.046**
Sex									
Male	Ref								
Female	1.238	0.46–3.32	0.671						
Weight loss 6 months prior to diagnosis									
Non	Ref								
< 10%	0.886	0.28–2.76	0.834						
≥ 10%	1.550	0.31–7.82	0.596						
BMI (kg/m2)	0.873	0.78–0.98	0.024**	0.867	0.77–0.98	0.022**	0.869	0.76–0.99	0.033**
Charlson comorbidity index	1.299	0.99–1.70	0.058						
TNM Stage									
I	Ref								
II	0.214	0.03–1.24	0.086						
III	0.240	0.04–1.51	0.129						
IV	0.273	0.05–1.37	0.115						
Frailty Fried criteria	1.916	1.28–2.87	0.002*				1.892	1.22–2.93	0.004*
Frailty GFI									
No	Ref								
Yes	1.884	0.69–5.12	0.214						
Frailty CGA									
No	Ref			Ref					
Yes	2.981	1.05–8.47	0.040**	2.537	0.83–7.76	0.103			

The first with multivariate analysis is conducted with Frailty CGA and the second with Frailty Fried criteria because of assumed multicollinearity

*BMI* body mass index, *GFI* groningen grailty indicator, *CGA* comprehensive geriatric assessment

^*^Correlation is significant at the 0.01 level (2-tailed)

^**^Correlation is significant at the 0.05 level (2-tailed)

**Table ****5** shows the univariate and multivariate logistic regression analyses with frailty, based on CGA, as the dependent variable. The univariate regression analysis with frailty as dependent variables distinguished CCI (OR 1.35 95% CI 1,03–1,76, *p* = 0.029), HSG (OR 0.92, 95% CI 0.87–0.97, *p* = 0.006), SMI (OR 0.89, 95% CI 0.83–0.96, *p* = 0.002), and sarcopenia (OR 2.98, 95% CI 1.05–8.47, *p* = 0.040) as significant variables for predicting frailty. These significant variables were subjected to a multivariate analysis. The first with sarcopenia and the second with HSG and SMI because of assumed multicollinearity. In the second only, SMI (OR 0.89, 95% CI 0.82–0.96, *p* = 0.003) remained significant.

**Table 5:** Univariate and multivariate logistic regression analysis for analyzing variables associated with frailty based on CGA in HNC patients

Frailty	Univariate analysis			Multivariate analysis					
	OR	95% CI	*p* value	OR	95% CI	*p* value	OR	95% CI	*p* value
Age (years)									
≤ 80	Ref								
> 80	2.533	0.98–6.55	0.055						
Charlson comorbidity index	1.350	1.03–1.76	0.029**	1.294	0.98–1.69	0.064	1.328	0.99–1.79	0.061
TNM Stage									
I	Ref								
II	0.825	0.15–4.50	0.824						
III	0.450	0.08–2.67	0.379						
IV	0.680	0.14–3.34	0.635						
HSG	0.922	0.87–0.97	0.006*				0.941	0.82–1.00	0.060
SMI	0.893	0.83–0.96	0.002*				0.887	0.82–0.96	0.003*
Sarcopenia									
No	Ref								
Yes	2.981	1.05–8.47	0.040**	2.494	0.85–7.34	0.097			

The first with multivariate analysis is conducted with Frailty CGA and the second with Frailty Fried criteria because of assumed multicollinearity

*BMI* body mass indexm, GFI groningen frailty indicator, CGA comprehensive geriatric assessment

^*^Correlation is significant at the 0.01 level (2-tailed)

^**^Correlation is significant at the 0.05 level (2-tailed)

## Discussion

In this study, the association between sarcopenia and frailty in 73 participants was retrospectively examined. Sarcopenia is associated with frailty defined with the CGA and Fried criteria, but not with the GFI frailty screening. Furthermore, the Fried criteria and BMI are significant predictors for sarcopenia. Frailty based on the CGA shows associations with the SMI and sarcopenia. Moreover, SMI shows to be a reliable predictor for frailty based on CGA. To our knowledge, this is the first study that examined the association between sarcopenia, as defined by low MF and low SMI, and frailty, as determined by CGA, in HNC patients.

With the aging of the global population, the incidence of frail and sarcopenic patients with HNC will increase. Understandings of the underlying interrelationship of sarcopenia and frailty are of great importance as they are both associated with adverse health outcome [7, 28]. Frailty and sarcopenia are important concepts in preventing physical dependence, as geriatrics are shifting towards identification of early stage of disability. Definitions of both sarcopenia and frailty are still developing, and both concepts clearly overlap in their physical aspects [11, 29]. Frailty is a pre-disability syndrome where an older person can be identified as being at risk when exposed to stressors associated with high risk for disability or needing to be hospitalized [30]. Two major frailty definitions exist. The physical phenotype of frailty (Fried) [26] and the multiple deficit model (Rockwood) [31]. CGA is the most appropriate way to detect frailty. Frailty is predisposed by advancing age in combination with physiological deterioration, especially a loss of muscle mass. So, sarcopenia is a major driver of frailty, because of decline of MF with low SMM. This increases the risk of falls, which can lead to loss of independence and disability. And low SMM increases the risk of comorbidity like diabetes mellitus and cardiovascular diseases by changing the body fat composition [30].

Studies using "physical" frailty as definition in examining the interrelationship with sarcopenia are suggested to have more overlap [29]. In this study, sarcopenic patients were more likely to be frail, according to the Fried criteria. Moreover, the Fried criterion was an independent predictor for sarcopenia. GFI was not associated with sarcopenia. Presumably because GFI uses also social, and psychological domains rather than only physically items like the Fried criteria. This confirms that “physical" frailty, like the Fried criteria, is more associated with sarcopenia than definitions based upon the multiple deficit model (Rockwood).

A previous retrospective study found a significant association between sarcopenia and frailty based on the G8 questionnaire (OR 0.76, 95% CI 0.6–0.89, *p* < 0.001) [10]. In that study, sarcopenia was based only on low SMI, so according to the EWGSOP-2 criterion, it was insufficient as sarcopenia which includes muscle function as well. Also, frailty screening was based on different screening questionnaires, i.e. G8, Timed Up and Go test, and Malnutrition Universal Screening Tool. In our study, SMI, but not the combination of low MF and low SMI (defined as sarcopenia by the EWGSOP-2), was independently associated with frailty based on CGA (OR 0.89, 95% CI 0.82–0.96, *p* = 0.003).

The suggestion that SMI could possibly be able to predict frailty, in particularly the physical part of frailty, in patients with HNC and is easier to use and implement, then a CGA or questionnaires to diagnose frailty is in accordance with the study of Zwart et al., although in our study, SMI was directly associated with CGA instead of the G8 frailty screening questionnaire [10].

Our study has some limitations. It was designed as a retrospective single-center study, with a limited number of included patients. Only patients with the available data on MF and SMI were included in the study. As it is more likely that MF parameters were examined for frail patients than for fit patients, this may have resulted in selection bias. Also, both CT and MRI imaging are used for the assessment of SMI, to maximize the number of patients that could be included. This could raise concerns but these two different imaging modalities show significant correlation in quantifying SMI when measured by CSA at the level of C3 [32]. At last, the majority (63%) of the patients had an oral cavity cancer. Other tumor locations as pharynx may cause more significant weight loss prior to diagnosis. In the present study, we used the commercially available software package SliceOmatic, but also free open-source software applications, e.g., 3D Slicer, can be used to assess CSA.

A strength of our study is that all of the muscle tissues were manually performed by a single researcher who was blinded for outcome regarding frailty and sarcopenia. Because an excellent inter-observer agreement for SMI measurement at the level of C3 was demonstrated, these SMI measurement findings can be used globally to tailor treatment according patients’ frailty [33].

In conclusion, there is an association between sarcopenia and frailty defined by CGA. Low muscle mass, based on SMI, may be able to predict some CGA domain outcomes in older patients with HNC and is easier to use and implement then a CGA. These findings should ideally be validated in a larger, prospective cohort study.
